# Ganglionated plexus ablation of the left atrium for refractory vasovagal syncope: Analysis of the Safety, Effectiveness and Related Factors

**DOI:** 10.1016/j.ipej.2025.12.017

**Published:** 2025-12-15

**Authors:** Nannan Ge, Yao Chen, Jie Han

**Affiliations:** The First Affiliated Hospital of Zhejiang University School of Medicine, Hang Zhou, China

**Keywords:** Vasovagal syncope, Ganglionated plexus ablation, Head-up tilt table test, Holter monitor

## Abstract

**Objective:**

To investigate the safety and efficacy of ganglionated plexus ablation of the left atrium for patients with refractory vasovagal syncope.

**Methods:**

From May 2019 to December 2023, 39 patients with refractory vasovagal syncope (VVS) underwent ganglionated plexus (GP) ablation at our institution. Associations between post-ablation VVS recurrence and factors including average heart rate on Holter monitoring, preoperative head-up tilt table test (HUTT), for GP identification methods, and sex were analyzed.

**Results:**

During the follow-up period 18.7 months, 30 of the 39 patients (76.9 % vs 23.1 %, p = 0.001) were symptom-free after GP ablation. The remaining 9 patients exhibited significant symptomatic improvement, with a marked reduction in the number of syncopal episodes (2.11 ± 1.27 vs 7.13 ± 3.57, p = 0.00). The mean heart rate after procedure (87.10 ± 11.22 bpm) was significantly higher than pre-procedures (70.33 ± 7.56 bpm, p = 0.00), indicating effective VVS control. Preoperative HUTT classifications and GP localization methods showed no significant association with recurrence, although female sex was associated with a higher likelihood of recurrent VVS. No procedure-related complication occurred.

**Conclusion:**

GP ablation of the left atrium is a safe and effective treatment for patients with refractory vasovagal syncope.

## 1 Introduction

Vasovagal syncope (VVS) is a transient loss of consciousness caused by a decrease in cardiac sympathetic excitability and an increase in vagal excitability, resulting in a decrease in heart rate and/or blood pressure. VVS is the most common cause of syncope at all ages, causing physical injuries and poor quality of life. The pathophysiological basis of VVS is the vagal hypertonicity [1]. The complex network of autonomic ganglionated plexus (GP) regulates the parasympathetic tone of the heart. Many researchers have attempted to reduce the tension of the parasympathetic nerves by performing GP ablation to alleviate the symptoms of VVS [2]. This current retrospective in nature study retrospectively reviewed 39 patients with refractory VVS who underwent GP ablation to assess the effectiveness and safety of this procedure.

## 2 Methods

### 2.1 Study population

39 patients with refractory VVS who underwent GP ablation in the left atrium at Zhejiang University School of Medicine First Hospital from May 2019 to December 2023 were enrolled. Inclusion criteria: (1) Met the diagnostic assessment for vasovagal syncope stipulated by the Expert Consensus on Diagnosis and Treatment of Vasovagal Syncope issued by the American Heart Rhythm Society in 2018; (2) Had a history of more than 3 syncopal episodes or at least one recurrence of syncope within 6 months before catheter ablation; (3) Underwent head-up tilt (HUT) test before surgery, and had a positive response. Exclusion criteria: (1) Syncope caused by structural heart disease, including but not limited to sinus node and atrioventricular dysfunction, hypertrophic cardiomyopathy, pulmonary hypertension, epileptic seizure, transient ischemic attacks, or drug-induced syncope; (2) Concurrent significant diseases, including myocardial infarction within 6 months, heart failure (NYHA classification III or IV), diabetes or cancer; (3) Previous history of cardiac surgery, catheter ablation, or permanent pacemaker implantation.

### 2.2 Head-up tilt test

After a 4-h fast, patients underwent HUTT consisting of two phases: a passive phase (70° tilt for 20 min ) and a provocative phase (sublingual nitroglycerin 0.25 mg if the passive phase was negative). Continuous ECG and blood pressure monitoring were performed. A positive response was defined as syncope or presyncope with hypotension (SBP <70 mmHg or DBP <40 mmHg) or bradycardia (HR < 40 bpm).

### 2.3 Holter monitor

24-h Holter monitoring was performed within one week before and after the procedure, with average heart rate recorded.

### 2.4 Mapping and GP localization

Patients were routinely disinfected and draped, and a femoral venous access was obtained under local anesthesia with 1 % lidocaine. A 6F decapolar steerable electrode catheter was then placed in the coronary sinus, followed by a 6F quadripolar electrode catheter positioned in the right ventricle apex. All patients had a routine conventional electrophysiological study before ablation.

**Fig. 1:**
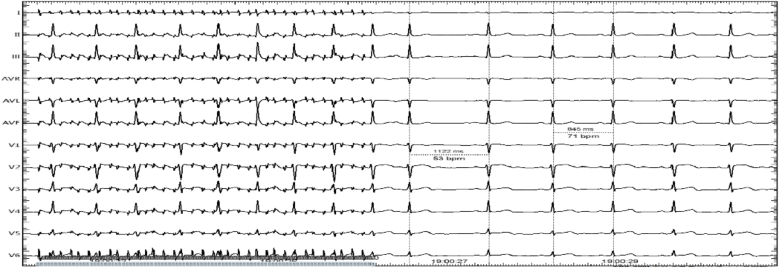
The first R-R interval after the termination of high-frequency stimulation is 1122 ms (53 beats/min), which is significantly longer than the normal R-R interval of 845 ms (71 beats/min), indicating a marked decrease in frequency.

Then the interatrial septum was punctured. The left atrium and four pulmonary veins were 3D mapped by the Biosense Webster system. An 8-mm-tip deflectable Cold saline ablation catheter was infused into the left atrium through the interatrial septum. The power and temperature limits were 40W and 40 °C,respectively. Two methods are available to localize the GP, the high-frequency stimulation (HFS) (50 Hz, 10–20V, 5 ms) method or the anatomical landmarks method. In the anatomical ablation group, the ablation endpoint was the disappearance of the vagal response in all GP anatomical regions. The followings are common GP locations: right anterior GP (RAGP) (anterior aspect of the right PV vestibulum); right inferior GP (RIGP) (located in the inferior posterior area around the root of the RIPV); left superior GP (LSGP) (usually located in the superolateral area around the root of the LSPV or between the anterior ridge and LSPV); left inferior GP (LIGP) (located in the inferior posterior area around the root of the LIPV ostium), and left lateral GP (LLGP) (located in the area around the ligament of Marshall). Then high-frequency stimulation (HFS) is utilized for ablation on the above common GP sites. During the HFS, HFS mapping was performed on the endocardial surface of the LA to search for positive response points and radiofrequency ablation, if a positive vagal response (VR) is recorded, i.e. transient ventricular asystole, AV block, or an increase in the mean RR interval for 50 % (Fig. 1), then it was marked as an ablation target [3].

### 2.5 Ablation of GP

Ablation was performed with irrigated catheters limited to 40W/40 °C. If a VR occurs during the ablation, it was considered an effective ablation target. Radiofrequency energy was delivered until vagal response disappearance (max 120 s per site). Activated clotting time was maintained at ≥300 s. Post-procedure anticoagulation consisted of rivaroxaban 10 mg daily for two months.

### 2.6 Follow-up

Holter monitor was repeated within a week post-ablation. Clinical follow-up via outpatient visits or telephone interviews was conducted at 3, 6, 12, and 24 months. Syncope or presyncope events were documented.

### 2.7 Statistical analysis

All data was analyzed using SPSS software version 23.0. The pre- and postoperative mean heart rates and the pre- and postoperative numbers of syncope episodes were compared using a paired sample *t*-test. Analysis of the syncope types in the recurrence group and the gender comparison in the recurrence group were performed using the chi-square test. A value of P < 0.05 was considered statistically significant for all statistical determinations.

### 2.8 Results

A total of 39 patients (aged 34.5 ± 17.7 years; male/female:10/29, p = 0.105) were included in this study. Based on preoperative HUTT, patients were classified as cardioinhibitory (33.3 %), vasodepressor (30.8 %), or mixed (35.9 %) (p = 0.926). GP localization was performed using anatomical landmarks in 32 (82.1 %) patients and HFS in 7 (17.9 %).

**Fig. 2:**
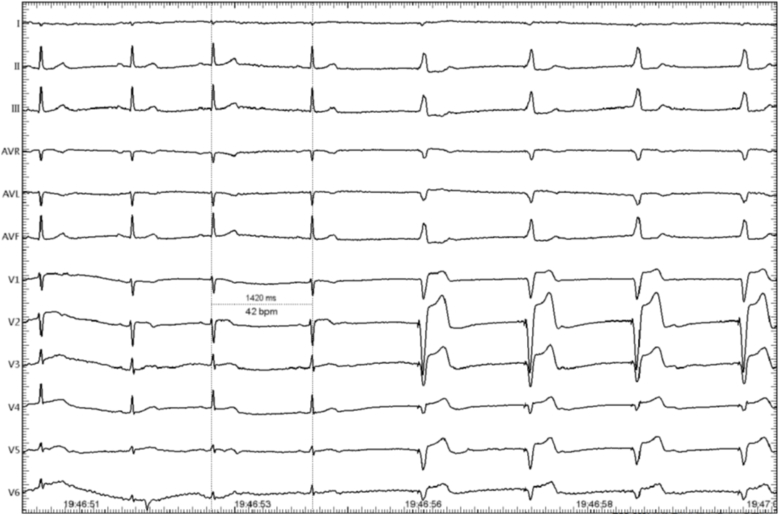
During the ablation process, a vagal response occurred, with a marked slowing of the ventricular rate. The quadripolar electrode catheter was burst at 40 beats per minute.

**Fig. 3:**
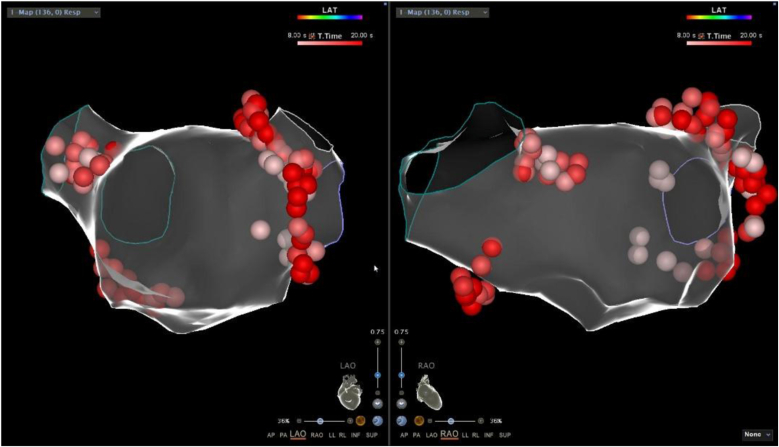
The three-dimensional image after GP ablation.

Fig. 2 shows an example of the radiofrequency application at the target sites. The map of the GP ablation targets is shown in Fig. 3.

Post-procedure heart rate significantly increased (87.10 ± 11.22 bpm vs. 70.33 ± 7.56 bpm, p < 0.001). This result demonstrated that GP ablation could reduce vagal tone through denervation.

During follow-up, 30 of the 39 patients (76.9 %) were free from syncope in comparison with 9 patients who still had symptoms (23.1 %, p = 0.001), suggesting effective VVS control following GP ablation. In the recurrence group, the number of syncope attacks (2.11 ± 1.27) after procedure was significantly lower than before (7.13 ± 3.57, p = 0.00), and we found that women were more likely to have syncope or presyncope recurrence (women vs men, 89 % vs 11 %, p = 0.039). Also, in the recurrence group, 8 (88.9 %) patients had ablation based on anatomical landmarks for GP localization, while only 1 (11.1 %) patient had HFS localization. For comparison, in the symptom-free group, 24 (80 %) patients had anatomical landmark methods, and 6 (20 %) patients had HFS methods (p = 0.48). This result suggested that GP localization method has no significant impact on the VVS control effectiveness. The follow-up results also showed that VVS classifications according to the preoperative HUTT have no significant impact on the recurrence outcome (Table 1, p = 0.97).

**Table 1:** VVS classifications according to the preoperative HUTT.

		Types			total
		cardioinhibitory	Vasodepressor	Mixed	
Recurrence	count	3	3	3	9
	%	33.3 %	33.3 %	33.3 %	100.0 %
Non-recurrence	count	10	9	11	30
	%	33.3 %	30.0 %	36.7 %	100.0 %

p = 0.97.

There were no procedure-related complications, including vascular access events, tamponade, pericarditis, or symptoms related to a delay in gastric emptying.

## 3 Discussion

VVS is a common type of syncope. Conventional management strategies for VVS include tilt training, oral rehydration therapy to expand blood volume, and pharmacologic treatments. Some researchers have proposed that pacemaker implantation may be beneficial for cardiac inhibitory subtypes of VVS [4]. However, this conclusion was challenged by a double-blind study conducted by Connolly et al., which demonstrated that pacemaker implantation did not reduce the risk of syncope in VVS patients [5]. Subsequent investigations revealed that the initial cardiac response to vagus nerve stimulation is an increase in myocardial contractility [6]. Building on this physiological mechanism, Biotronik developed a closed-loop stimulation (CLS) pacemaker capable of initiating pacing prior to the onset of bradycardia and hypotension. This device utilizes intracardiac impedance measurements to detect changes in myocardial contractility, thereby increasing heart rate and cardiac output to prevent syncope [7,8]. Nevertheless, clinical evidence supporting the efficacy of CLS remains limited.

GP ablation has emerged as a novel and promising intervention for VVS, first introduced by Pachon et al. [9]. This method is based on the observation that patients with atrial fibrillation (AF) who underwent pulmonary vein isolation through catheter ablation and then autonomic nerve ablation, would have an improved AF treatment outcome and a lower recurrence rate ^[^ [[10], [11], [12]]^]^. Pachon et al. hypothesized that ablating the ganglionated plexi at the root of the pulmonary veins using radiofrequency ablation could treat patients with VVS caused by excessive vagal activity. Subsequently, Pachon et al. conducted a study on 21 VVS patients who underwent GP ablation. All patients showed improvement in their syncope symptoms at a follow-up of 9.2 months, with no reported complications [9]. Subsequently, Yan et al. successfully performed targeted GP ablation in the left atrium in 10 patients with recurrent VVS and positive head-up tilt tests (HUTT) [13]. In 2016, T. Aksu et al. reported GP ablation on 21 patients with cardioinhibitory VVS, with no recurrent syncope at one-year of follow-up [14]. In 2020, the same group published a single-center experience involving 51 patients with refractory VVS; after a median follow-up of 11 months, only 3 patients experienced recurrent syncope, yielding a success rate of 86.2 % [15]. Our study contributes the largest cohort to date, comprising 39 VVS patients, with the longest follow-up period (18.7 months). Our results support that GP ablation is a safe and effective treatment for patients with recurrent VVS who have failed conventional conservative management.

It is believed that VVS patients with cardiac inhibitory and mixed type were more suitable for GP ablation [13,14,16]. However, our analysis revealed no significant difference in recurrence between VVS types (p = 0.97), indicating that HUTT-based classification may not aid in patient selection for GP ablation. This finding is consistent with the management guidelines for syncope patients issued by the American Heart Rhythm Society, which states that the classification of VVS by the HUTT has no guiding significance for its treatment outcome [17]. In contrast, T. Aksu's study reported that GP ablation showed the best outcomes for patients with refractory cardiac inhibitory type of VVS who were under 60 years of age [18]. On the other hand, Yan et al. observed the lowest event-free rate after cardioneuroablation in vasodepressive-type VVS [19]. However, 55 % of patients showed no syncope/pre-syncope recurrence after GP ablation in vasodepressive VVS, and the syncope/pre-syncope burden was significantly reduced after the procedure might provide that GP ablation might benefit vasodepressive type. Additionally, GP ablation might alleviate vasodepression through afferent nerve denervation. Baroreflex dysfunction has been implicated in the pathophysiology of VVS [20], and GP ablation impaired both the efferent and afferent nerve of the baroreflex in the heart. We may need longer follow-up to confirm these results. Heart rate variability (HRV) and deceleration capacity (DC) are non-invasive parameters derived from ECG using specialized algorithms. HRV, based on RR interval variability, is often reduced in VVS patients. DC, derived from HRV analysis, values greater than 7.5 ms suggest heightened parasympathetic activity ^[^ [21]、 [22]^]^. In future studies DC and HRV can be better substitutes for monitoring response to ablation on follow-up. In our study, the GPs were located by either anatomical landmarks or HFS methods. The results indicated no significant difference in recurrence rates between the two methods. Yan et al. similarly reported that the method of GP localization—anatomical or HFS-guided—did not affect clinical outcomes [16,23,24]. Moreover, Wei et al. found that anatomical ablation shortened procedure and fluoroscopy times while achieving success rates comparable to HFS-guided ablation in preventing syncope recurrence [25].

Additionally, we observed that female patients had a higher recurrence rate than male. The underlying reasons for this gender disparity remain unclear. Romme et al. investigated gender influences on reflex syncope and found that emotional or pain triggers and prolonged standing were reported similarly by both sexes [26]. The emotional triggers may account for the sex differences in recurrence rate. However, more data and long-term clinical follow-up are needed for further clarification on the impact of gender on the success rate of GP ablation. For younger patients, pacemaker implantation may impose long-term social and psychological burdens, whereas GP ablation could circumvent these concerns [27]. This study has certain limitations. For example, the absence of a sham surgery or placebo as a control may lead to an overestimation of the therapeutic effect of ganglionated plexus ablation of the left atrium for refractory vasovagal syncope.

## 4 Conclusion

GP ablation of the left atrium is a safe and effective treatment for patients with refractory vasovagal syncope. The main limitations of this study are the limited number of patients enrolled and the relatively short follow-up period. We will continue to enroll more patients and follow up with all patients in the longer term in our future clinical study.

## Funding

Have no funding.

## Declaration of competing interest

None of the authors have any financial or scientific conflicts of interest with regard to the research described in this manuscript.

## References

[1] Chen-Scarabelli C., Scarabelli T.M. (2004 Aug 7). Neurocardiogenic syncope. BMJ.

[2] Yao Y., Shi R., Wong T. (2012 Apr). Endocardial autonomic denervation of the left atrium to treat vasovagal syncope: an early experience in humans. Circ Arrhythm Electrophysiol.

[3] Xu L., Zhao Y., Duan Y. (2022 Sep 13). Clinical efficacy of catheter ablation in the treatment of vasovagal syncope. Clin Med.

[4] Sutton R., Brignole M., Menozzi C. (2000 Jul 18). Dual-chamber pacing in the treatment of neurally mediated tilt-positive cardioinhibitory syncope: pacemaker versus no therapy: a multicenter randomized study. The Vasovagal Syncope International Study (VASIS) investigators. Circulation.

[5] Connolly S.J., Sheldon R., Thorpe K.E. (2003 May 7). VPS II investigators. Pacemaker therapy for prevention of syncope in patients with recurrent severe vasovagal syncope: Second Vasovagal Pacemaker Study (VPS II): a randomized trial. JAMA.

[6] Kosinski D.J., Grubb B.P., Wolfe D.A. (2004 Oct). Permanent cardiac pacing as primary therapy for neurocardiogenic (reflex) syncope. Clin Auton Res.

[7] Osswald S., Cron T., Grädel C. (2000 Oct). Closed-loop stimulation using intracardiac impedance as a sensor principle: correlation of right ventricular Dp/dtmax and intracardiac impedance during dobutamine stress test. Pacing Clin Electrophysiol.

[8] Drago F., Silvetti M.S., De Santis A. (2005 Mar). Beat-to-beat heart rate adaptation in pediatric and late adolescent patients with closed loop rate-responsive pacemakers. Pacing Clin Electrophysiol.

[9] Pachon J.C., Pachon E.I., Pachon J.C. (2005 Jan). "Cardioneuroablation"--new treatment for neurocardiogenic syncope, functional AV block and sinus dysfunction using catheter RF-ablation. Europace.

[10] Lemola K., Chartier D., Yeh Y.H. (2008 Jan 29). Pulmonary vein region ablation in experimental vagal atrial fibrillation: role of pulmonary veins versus autonomic ganglia. Circulation.

[11] Choi E.K., Zhao Y., Everett T.H. (2017 Dec). Ganglionated plexi as neuromodulation targets for atrial fibrillation. J Cardiovasc Electrophysiol.

[12] Pappone C., Santinelli V., Manguso F. (2004 Jan 27). Pulmonary vein denervation enhances long-term benefit after circumferential ablation for paroxysmal atrial fibrillation. Circulation.

[13] Yao Y., Shi R., Wong T. (2012 Apr). Endocardial autonomic denervation of the left atrium to treat vasovagal syncope: an early experience in humans. Circ Arrhythm Electrophysiol.

[14] Aksu T., Golcuk E., Yalin K. (2016 Jan). Simplified cardioneuroablation in the treatment of reflex syncope, functional AV block, and sinus node dysfunction. Pacing Clin Electrophysiol.

[15] Aksu T., Guler T.E., Bozyel S. (2020 Sep 1). Usefulness of post-procedural heart rate response to predict syncope recurrence or positive head up tilt table testing after cardioneuroablation. Europace.

[16] Yao Y., Shi R., Wong T. (2012 Apr). Endocardial autonomic denervation of the left atrium to treat vasovagal syncope: an early experience in humans. Circ Arrhythm Electrophysiol.

[17] Shen W.K., Sheldon R.S., Benditt D.G. (2017 Aug 1). 2017 ACC/AHA/HRS guideline for the evaluation and management of patients with syncope: a report of the American college of Cardiology/American heart association task force on clinical practice guidelines and the heart rhythm society. Circulation.

[18] Aksu T., Chung M.K. (2024 Apr). Cardioneuroablation for cardioinhibitory vasovagal syncope: Rationale, approaches, and its role in long-term management. Curr Cardiovasc Risk Rep.

[19] Tu B., Lai Z.H., Chen A.Y. (2024 Jun 28). Effectiveness of cardioneuroablation in different subtypes of vasovagal syncope. Geriatr Cardiol.

[20] Kaufmann H., Norcliffe-Kaufmann L., Palma J.A. (2020 Jan 9). Baroreflex dysfunction. N Engl J Med.

[21] Chakraborty P., Chen P.S., Gollob M.H. (2024 Apr). Potential consequences of cardioneuroablation for vasovagal syncope: a call for appropriately designed, sham-controlled clinical trials. Heart Rhythm.

[22] Aksu T., Po S.S. (2024 Jan). How to perform cardioneuroablation for vasovagal syncope and functional bradycardia. Heart Rhythm.

[23] Zheng L., Sun W., Liu S. (2020 Dec). The diagnostic value of cardiac deceleration capacity in vasovagal syncope. Circ Arrhythm Electrophysiol.

[24] Pachon J.C., Pachon E.I., Aksu T. (2023 Mar 21). Cardioneuroablation: where are we at?. Heart Rhythm O2.

[25] Sun W., Zheng L., Qiao Y. (2016 Jul 8). Catheter ablation as a treatment for vasovagal syncope: long-term outcome of endocardial autonomic modification of the left atrium. J Am Heart Assoc.

[26] Romme J.J., van Dijk N., Boer K.R. (2008 Jun). Influence of age and gender on the occurrence and presentation of reflex syncope. Clin Auton Res.

[27] Joza J., Alturki A., Anglesio V. (2024 Feb 13). Cardioneuroablation as a strategy to prevent pacemaker implantation in young patients with vasovagal syncope. Int J Cardiol Heart Vasc.

